# Supplementary figures and images for: Simulated Microgravity Created Using a Random Positioning Machine Induces Changes in the Physiology of the Fusarium solani Species Complex
Source: Microorganisms. 2022 Nov 16;10(11):2270. doi: 10.3390/microorganisms10112270 (PMC9696954; doi:10.3390/microorganisms10112270)

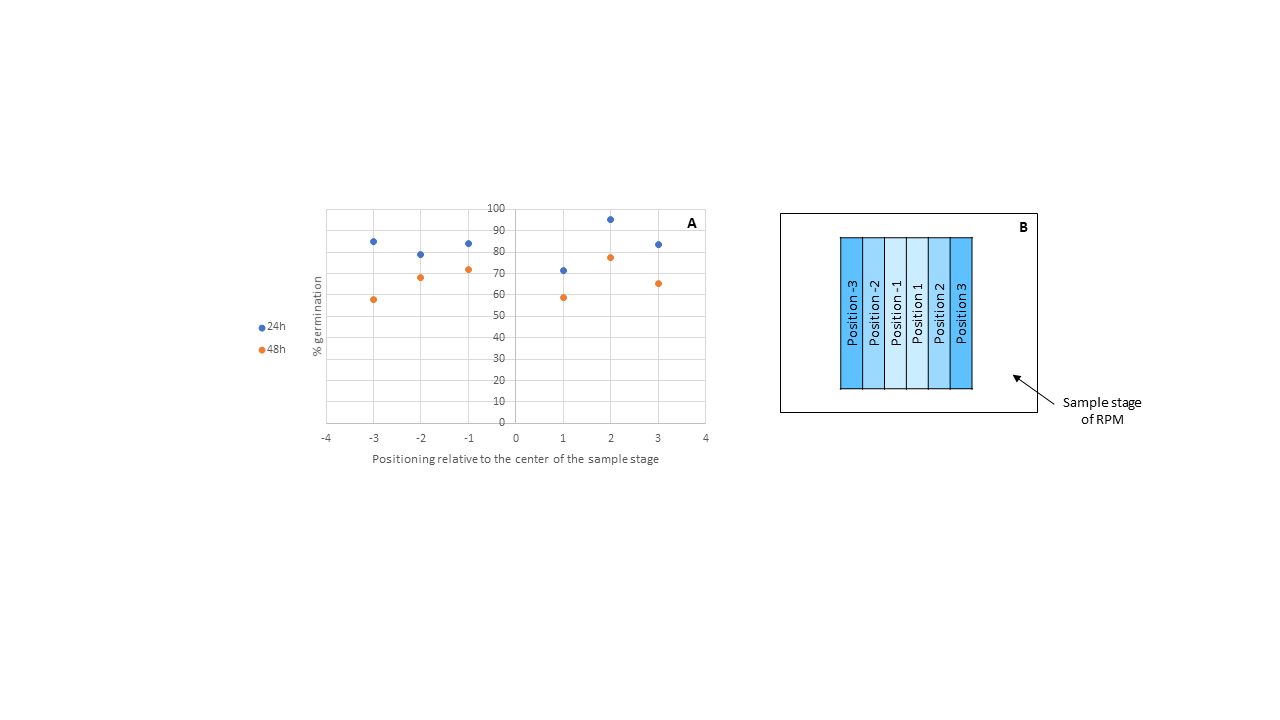

Supplement: Supplementary file 1 [file microorganisms-10-02270-s001.zip › microorganisms-2005133-Figure S1.tif]
